# Intragastric free cancer cells may be attached to automatic staplers during anastomosis in patients with gastric cancer

**DOI:** 10.1186/s12957-023-03285-2

**Published:** 2024-01-04

**Authors:** Atsuko Ohki, Taisuke Takagi, Yohei Kojima, Masanao Tsurumi, Yoshikazu Hashimoto, Hirohisa Takeuchi, Hiroshi Kamma, Yoshihiro Sakamoto, Eiji Sunami, Nobutsugu Abe

**Affiliations:** 1 Department of Gastroenterological and General Surgery, Kyorin University Faculty of Medicine, 6-20-2 Shinkawa, Mitaka, Tokyo, 181-8611 Japan; 2 Nasu Institute of Medical Sciences, 2-5 Daikokuchou, Nasushiobara, Tochigi, 325-0046 Japan

**Keywords:** Gastric cancer, Surgical staplers, Gastrectomy, Anastomosis, Recurrence

## Abstract

**Background:**

Automatic staplers are often used to reconstruct the digestive tract during surgeries for gastric cancer. Intragastric free cancer cells adhering to automatic staplers may come in contact with the laparoscopic port area and progress to port site recurrence. This study aimed to investigate the presence/absence of cancer cells adhering to automatic staplers during gastric cancer surgery using cytological examinations. We further determined the positive predictive clinicopathological factors and clinical implications of free cancer cells attached to automatic staplers.

**Methods:**

This study included 101 patients who underwent distal gastrectomy for gastric cancer. Automatic staplers used for anastomosis in gastric cancer surgeries were shaken in 150 ml of saline solution to collect the attached cells. Papanicolaou stains were performed. We tested the correlation between cancer-cell positivity and clinicopathological factors to identify risk factors arising from the presence of attached cancer cells to the staplers.

**Results:**

Based on the cytology, cancer cells were detected in 7 of 101 (6.9%) stapler washing fluid samples. Univariate analysis revealed that circular staplers, type 1 tumors, and positive lymph nodes were significantly associated with higher detection of free cancer cells adhering to staplers. No significant differences in other factors were detected. Of the seven cases with positive cytology, one developed anastomotic recurrence.

**Conclusions:**

Exfoliated cancer cells adhered to the automatic staplers used for anastomoses in 6.9% of the staplers used for distal gastrectomies in patients with gastric cancer. Staplers used for gastric cancer surgeries should be handled carefully.

## Background

In gastric cancer surgeries, automatic staplers such as linear or circular staplers are often used to reconstruct the digestive tract. The automatic stapler is inserted into the gastric lumen for the anastomosis. We previously demonstrated that gastric cancer cells can be largely exfoliated from the tumor surface into the gastric lumen by endoscopic irrigation [1]. Thus, intragastric free cancer cells can be adhered to automatic staplers during surgery. If intragastric free cancer cells adhere to the automatic stapler, these free cancer cells may come in contact with the laparoscopic port area and progress to port site recurrence. Port site recurrences develop in 0.4–11% of laparoscopic gastrectomies for gastric cancer [2]. While using an automatic stapler during gastric reconstruction, intragastric free cancer cells may come in contact with the anastomotic line and cause anastomotic recurrence. Anastomotic recurrences after gastrectomy have been reported in several studies, and these recurrences may be related to the implantation of free cancer cells [3, 4]. However, the role of free cancer cells attached to an automatic stapler during gastric cancer surgery has not been investigated. Moreover, cancer cells adhering to automatic staplers during gastric cancer surgery have rarely been studied. This study aims to determine positive predictive clinicopathological factors and the clinical consequences of free cancer cells adhered to automatic staplers.

## Methods

### Patients

Between March 2020 and March 2023, 101 patients who underwent distal gastrectomies of gastric cancers at the Department of Gastroenterological and General Surgery, Kyorin University Hospital were enrolled in the study. All patients underwent open, laparoscopic, or robot-assisted distal gastrectomies and lymph node dissections. The study population included 62 male and 39 female patients, with a mean age of 73 years (range 35–90 years). Clinicopathological parameters were determined based on the Japanese Classification of Gastric Carcinoma established by the Japanese Research Society for Gastric Cancer [5]. Written informed consent was obtained from every patient. The study was approved by the Institutional Review Board of Kyorin University.

### Sample collection and cytological procedures

**Fig. 1:**
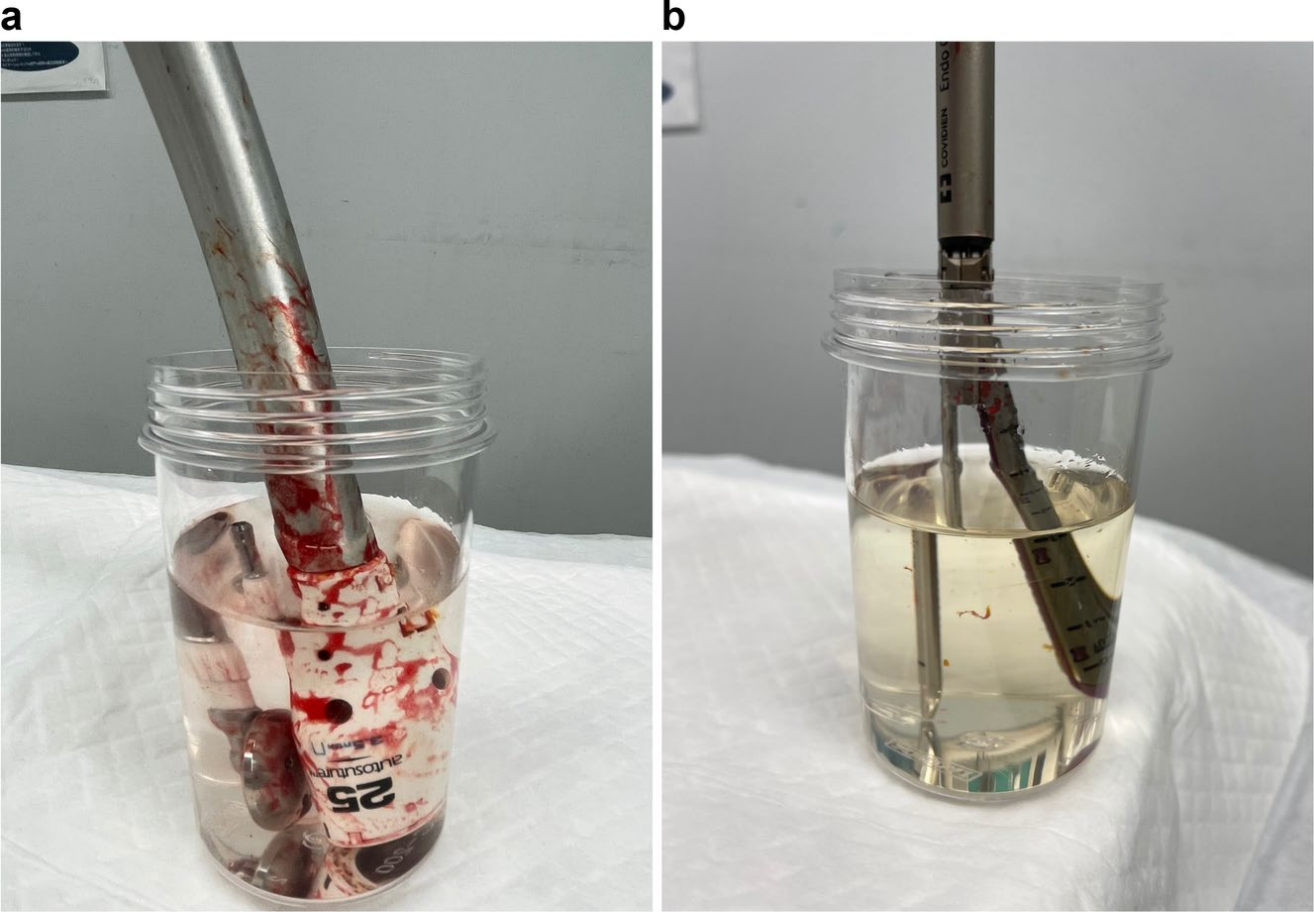
Sample collection from stapler cytology. An automatic stapler used for anastomosis in gastric cancer surgery was shaken in 150 ml of saline solution to collect the attached cells

For each patient, an anastomosis was performed using a circular or linear automatic stapler after the gastric cancer was resected. The automatic stapler was then shaken in 150 ml of saline solution to collect the attached cells (Fig. 1). The fluid sample was immediately centrifuged for 5 min at 2000 rpm, and the cells were stored in cell preservation media. The collected cells were attached to glass slides and stained with Papanicolaou and Giemsa stains. Normal glandular cells, squamous cells, erythrocytes, granulocytes, food debris, and exfoliated cancer cells were identified. The exfoliated cancer cells appeared as single cells or lump formations, with large irregular nuclei and increased nuclear-to-cytoplasm ratios. Experienced cytologists and pathologists, who were blinded to all clinical information, classified the cytological findings into five classes (Class I–V), and Class III or higher cells were selected. Class III and above cells were compared with the histology of the resected main lesions, and very similar findings were defined as positive cytology. The correlations between cancer-cell positivity and clinicopathological factors were analyzed to identify risk factors for the presence of attached cancer cells.

### Statistical analyses

All statistical analyses were performed using SPSS Ver. 27.0.1. Clinicopathological characteristics were compared using Fisher’s exact probability tests, chi-square tests, or Student’s *t*-tests, where appropriate. Differences were considered significant at *p* < 0.05.

## Results

Patient characteristics are shown in Table 1. Of the 101 patients included in the study, 62 were men and 39 were women. The mean age of the patients was 73 (range 35–90) years. All patients underwent distal gastrectomies with lymph node dissections, including 33 open gastrectomies, 51 laparoscopic gastrectomies, and 17 robotic-assisted gastrectomies. Reconstruction was achieved using the Billroth I method in 98 patients and the Roux-en Y method in 3 patients. Circular staplers were used in 64 patients and linear staplers were used in 37 patients. According to the Japanese Classification of Gastric Carcinoma, 58 patients had stage I, 26 patients had stage II, and 17 patients had stage III cancers.

**Table 1:** Patient characteristics

Variable		All (*N* = 101)	
**Sex**	Male	62	
	Female	39	
**Age (mean)**		73	35–90
**Operation**	Open	33	
	Laparoscopy	51	
	Robotic-assisted laparoscopy	17	
**Lymph node dissection**	D0, D1	17	
	D1+	71	
	D2	13	
**Reconstruction**	B-I	98	
	R-Y	3	
**Stapler**	Circular	64	
	Linear	37	
**Tumor location**	M	44	
	L	57	
**Macroscopic type**	0-I, IIa	26	
	0-IIb	2	
	0-IIc	32	
	Type 1	7	
	Type 2, 3	29	
	Type 4, 5	6	
**Tumor size (mm)**		44	5–180
**Depth of invasion**	m	24	
	sm	27	
	mp	18	
	ss	19	
	se, si	13	
**Histological grade**	Differentiated	57	
	Undifferentiated	44	
**Lymphatic involvement**	Positive	52	
	Negative	49	
**Vessel involvement**	Positive	60	
	Negative	41	
**Lymph node metastasis**	N0	66	
	N1	14	
	N2	13	
	N3a	5	
	N3b	3	
**CY**	CY0	26	
	CY1	2	
	CYX	73	
**Stage**	IA, IB	58	
	IIA, IIB	26	
	IIIA, IIIB	17	
**Stapler washing cytology**	Positive	7	
	Negative	94	
**Postop cytology**	Positive	3	
	Negative	98	

*m* mucosa, *sm* submucosal, *mp* muscularis propria, *ss* subserosa, *se* serosa, *si* invading adjacent organs

Seven of the 101 cases (6.9%) were positive for cancer cells in the stapler wash. The relationships between clinicopathological factors and positive cancer cells are shown in Table 2. Univariate analysis revealed that circular stapler, type 1 tumor, and positive lymph nodes were significantly associated with the detection of free cancer cells. No significant relationships between the other clinicopathological factors and positive cancer cells in the stapler wash cytology were detected.

**Table 2:** Relationships between clinicopathological factors and positive cancer cells

		Cytology		
		Positive (*N*=7)	Negative (*N*=94)	*P*
**Gender**	Male	5	57	N.S.
	Female	2	37	
**Age**		79.9	72.6	N.S.
**Operatin**	Open	4	29	N.S.
	Laparoscpy	3	48	
	Robott assisted	0	17	
**Lymph node dissection**	D0, D1	3	14	N.S.
	D1+	3	68	
	D2	1	12	
**Reconstruction**	B-I	7	91	N.S.
	R-Y	0	3	
**Stapler**	Circular	7	57	0.04
	Linear	0	37	
**Tumor location**	M	5	39	N.S.
	L	2	55	
**Macroscopic type**	0-I, IIa	1	24	0.004
	0-Iib, Iic	1	33	
	Type 1	3	4	
	Type 2, 3	2	27	
	Type 4, 5	0	6	
**Tumor size (mm)**		49	43.6	N.S.
**Depth of invasion**	m	0	24	N.S.
	sm	3	24	
	mp	1	17	
	ss	1	18	
	se, si	2	11	
**Pathological type**	Differentiated	4	53	N.S.
	Undifferentiated	3	41	
**Lymphatic involvement**	Positive	6	46	N.S.
	Negative	1	48	
**Vessel involvement**	Positive	5	55	N.S.
	Negative	2	39	
**Lymph node metastasis**	Positive	5	30	0.047
	Negative	2	64	
**CY**	CY0	4	22	N.S.
	CY1	0	2	
	CYX	3	70	
**Stage**	IA, IB	2	56	N.S.
	IIA, IIB	3	22	
	IIIA, IIIB	2	16	

### Positive cytology cases

**Fig. 2:**
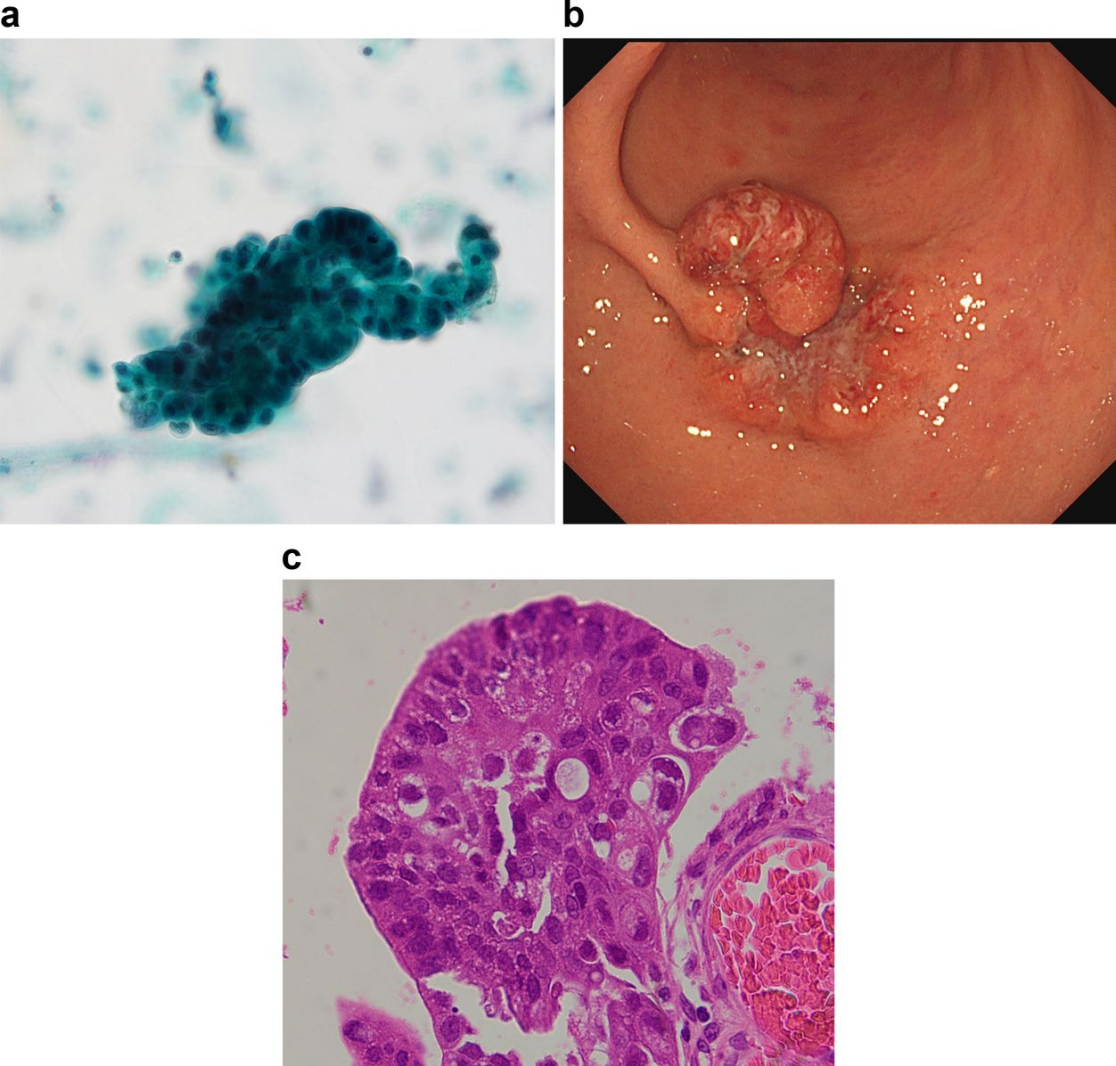
Positive cytology case 1. **a** Papanicolaou staining cytology detected clumps of free cancer cells. **b** Type 0-I + IIa; IIc gastric cancer in the middle of the stomach. The tumor size was 40 mm. **c** The patient had moderately differentiated adenocarcinoma invading the submucosa, positive lymphatic invasion, positive lymph node metastasis, and p Stage IIA

Two positive cytology cases are presented. In case 1, lumps of free cancer cells were detected in the stapler wash fluid (Fig. 2a). This case was early gastric cancer in the middle of the stomach (Fig. 2b). Pathological findings showed macroscopic tumor type 0-I + IIa + IIc, moderately differentiated adenocarcinoma (Fig. 2c), a tumor size of 40 mm, deep invasion of the submucosal, positive lymphatic involvement, positive lymph node metastasis (5/75), and pStage IIA. Open distal gastrectomy was performed and a circular stapler was used in the reconstruction.

**Fig. 3:**
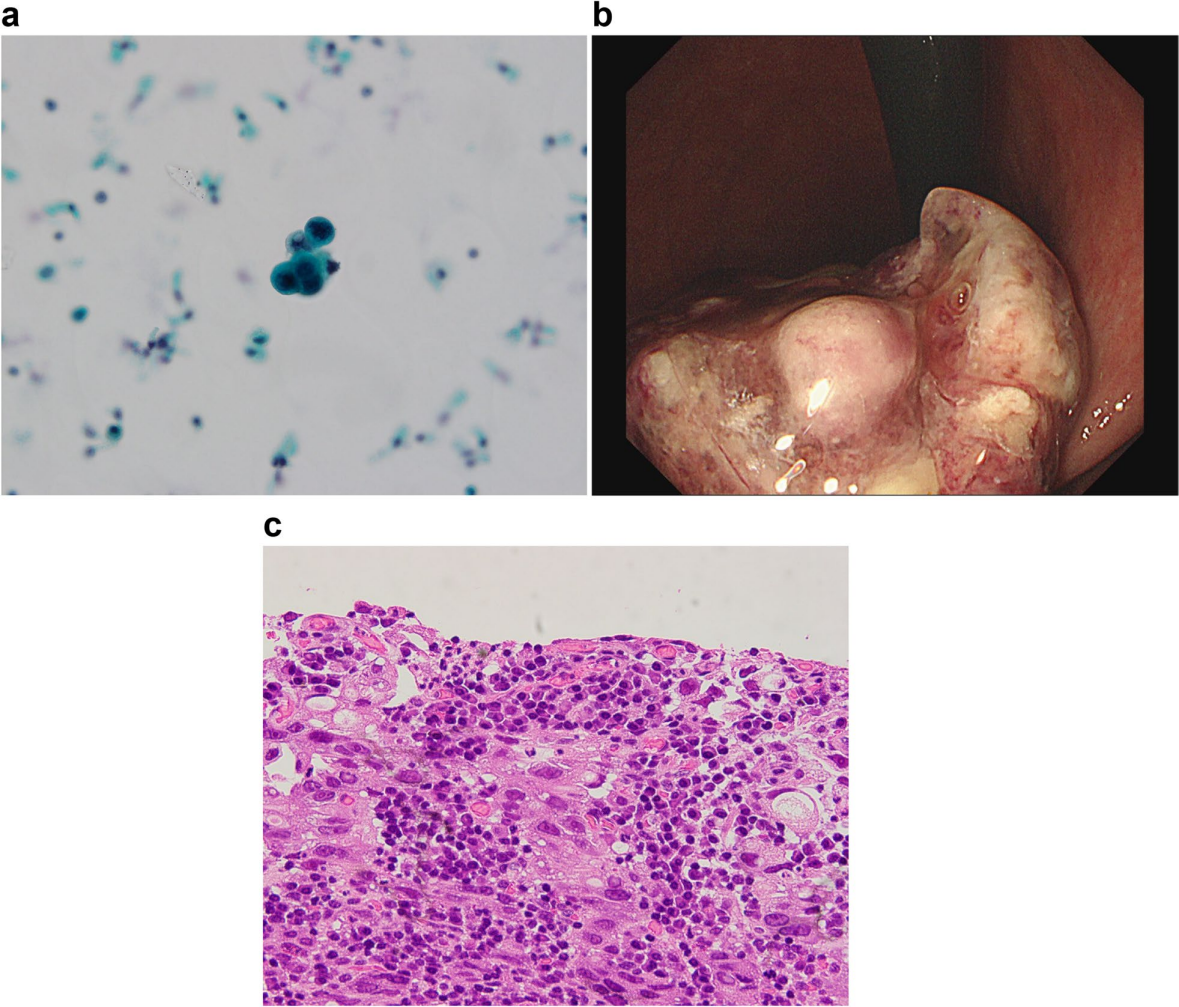
Positive cytology case 2. **a** Papanicolaou staining cytology detected single cancer cells. **b** Type 2 gastric cancer in the middle of the stomach. The tumor size was 75 mm. **c** The patient had poorly differentiated adenocarcinoma invading the muscular propria, positive lymphatic and vessel invasion, positive lymph node metastasis, and p Stage IIB

In case 2, single-cell fragments were detected in the stapler wash fluid (Fig. 3a). This case was advanced gastric cancer in the middle of the stomach. Pathological findings showed macroscopic type 2 (Fig. 3b), poorly differentiated adenocarcinoma (Fig. 3c), a tumor size of 73 mm, invasion of the muscular propria, positive lymphatic and vessel involvement, positive lymph node metastasis (6/41), and pStage IIB. A laparoscopic distal gastrectomy was performed and reconstruction was accomplished using a circular stapler. The cytology of the cells from the stapler wash was very similar to the histology of the main lesion.

### Follow-up outcomes

**Fig. 4:**
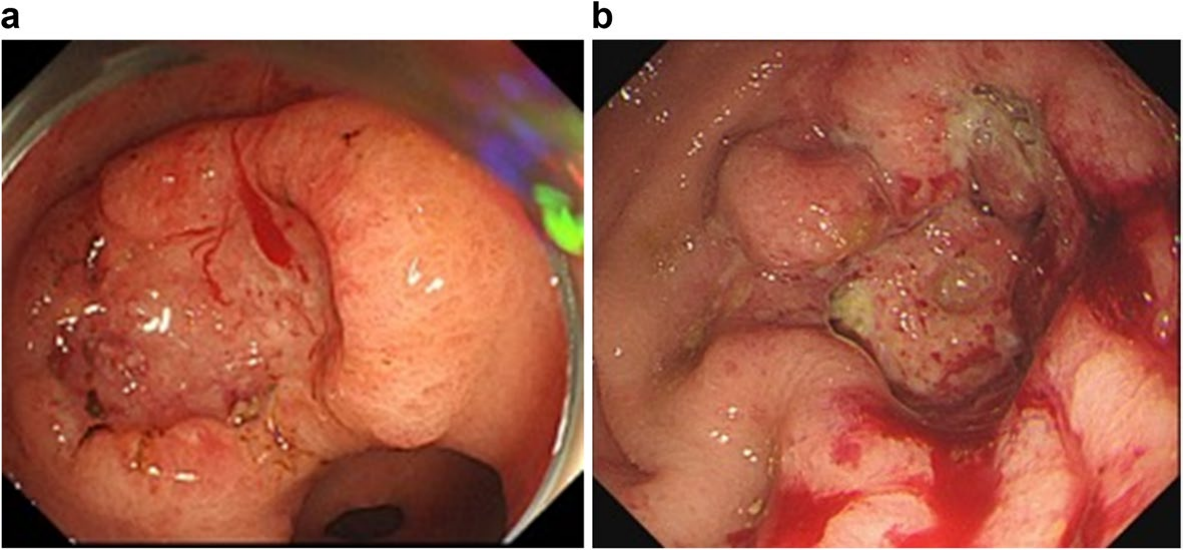
Case of anastomotic recurrence with cytology similar to the automatic stapler. **a** Advanced gastric cancer in the L region with depth and poorly differentiated carcinoma. **b** A full circumferential tumor recurrence was found to involve the stapler at the anastomosis

Postoperative follow-up ranged from 5 to 41 months. Anastomotic recurrence occurred in one case during the follow-up period. This patient had positive cytology for the automatic stapler cell wash. This patient had advanced carcinoma in the L region with invasion of the serosa, poorly differentiated carcinoma, positive lymphovascular invasion, and positive lymph node metastasis (Fig. 4a). An open distal gastrectomy and Billroth 1 reconstruction were performed in this patient. No cancer was detected at the resection margin. Nine months after the gastrectomy, stenosis, and ulcer formation were observed at the anastomosis. A biopsy revealed cancer cells similar to the previous gastric cancer. A metal stent insertion was performed for the anastomotic recurrence due to worsened chronic respiratory disease (Fig. 4b).

Of the other six patients with cancer cells in the stapler wash, five showed no anastomotic/peritoneal recurrences, but one died of multiple liver metastases and peritoneal dissemination.

## Discussion

Our results showed that exfoliated cancer cells sometimes adhere to automatic staplers used for anastomosis. Cancer cells were detected in the stapler wash in 6.9% of the gastrectomy cases. Univariate analysis revealed that circular stapler use, type 1 tumor, and positive lymph node metastasis were significantly associated with the detection of free cancer cells.

We previously reported that free cancer cells are exfoliated from the main tumor surface and fall into the stomach lumen when the surface of gastric cancer is washed during endoscopy [1]. Free cancer cells can detach from the tumor surface of gastric cancers, even with the minimal stimulation of washing. In our previous study, free cancer cells were shed into the gastric lumen from the main lesion in 50% of early-stage cancers and 78% of advanced cancers [1]. Tumor size more than 20 mm, deeper invasion into the gastric wall, and positive lymphovascular invasion were significantly associated with a higher detection rate of free cancer cells in the previous study. However, these factors were not significantly associated with the detection of cancer cells in the stapler wash fluid in the present study.

The presence of intraluminal tumor cells in colorectal cancer patients is well-known [6]. Many studies demonstrated the ability of intraluminal tumor cells to implant in colorectal anastomoses. Implantation of intraluminal exfoliated rectal cancer cells is one mechanism for anastomotic recurrence after rectal surgery [7]. In a previous study, free cancer cells were detected in the circular stapler and rectal tissues in 90% of cases if rectal cleansing was not performed before anastomosis of rectal cancer [8]; two cases developed local recurrence in that study. Therefore, intraoperative rectal washing before anastomosis is advised for rectal cancers. Ikehara et al. reported that 20% of automatic staplers used for anastomosis of colorectal cancer had attached free cancer cells but no anastomotic recurrences were detected. In the Ikehara study, linear stapler cartridges used for anastomotic sites in colon cancer were examined. Preoperative bowel preparation with polyethylene glycol solution significantly reduced the detection rate of free cancer cells [9].

Murata et al. [10] reported that viable cancer cells were detected by lavage cytology in the remnant stomach before anastomosis in 23.2% of cases. However, an anastomotic recurrence resulting from the implantation of such viable cancer cells after gastric cancer surgery is extremely rare [1, 2, 11–15]. Anastomotic recurrences using a linear stapler have also been reported, including an anastomotic recurrence after delta anastomosis to treat early gastric cancer [3]. Suture recurrences at the J-pouch site have also been reported [4, 14, 15] in reconstructions after total gastrectomy or proximal gastrectomy, and implantation of cancer cells was suspected. In our study, cancer cells from the stapling device may have been implanted in at least one case. The possibility that anastomotic recurrence was caused by cancer cells attached to the automatic stapler could not be ruled out. New automatic suturing devices must be used in each phase of the surgical procedure for gastric cancer. Polychronidis et al. [11] suggest that the stapler cartridges should be changed for new ones after each use. On the other hand, hand-sewn anastomosis is expected to result in less attachment of free cancer cells and tissue biting compared to anastomosis with an automatic stapler. However, the possibility of an implantation of free cancer cells in an anastomotic site even in hand-sewn anastomosis cannot be ruled out; therefore, attention should be paid to this potential phenomenon. Compared to colorectal cancer, the probability of anastomotic recurrence of gastric cancer is low, and surgeons know this empirically. Therefore, surgeons do not routinely perform prophylactic procedures against free cancer cells, such as gastric lavage and cleansing before anastomosis. Experimentally, distilled water exposure can rapidly increase cancer-cell volume, followed by cell rupture. The cytocidal effect of hypotonic stress on tumor cells has been demonstrated in various malignancies [16, 17]. To prevent recurrence due to cancer cells attached to the automatic staplers during gastric cancer surgery, drinking hypo-osmotic fresh water before surgery or stomach lumen lavage with hypo-osmotic water through the nasogastric tube during surgery may be helpful. It was reported that surgical manipulation may release free cancer cells from the stomach lumen or lymphatic vessel stalks opened during gastric cancer surgery into the abdominal cavity. It was suggested that free cancer cells attaching to the automatic staplers may also be involved in this increase in the number of free cancer cells in the abdominal cavity [18]. Abdominal lavage with low-osmotic distilled water is also effective for freeing cancer cells in the abdominal cavity [17].

In this study, circular staplers were associated with a higher frequency of cancer cells compared with linear staplers. Cancer cells adhered to circular staplers more frequently, but this may be due to the bigger tissue-biting areas. Anastomotic recurrences with linear staplers have also been reported, and similar attention should be paid. Further study is needed to clarify this issue.

## Conclusions

In conclusion, we revealed that exfoliated cancer cells adhered to automatic staplers used for anastomosis in approximately 6.9% of gastrectomy cases. The use of distilled water to lavage the stomach and changing cartridges in the automatic suture device may help prevent cancer-cell adherence and recurrence. Special attention should be paid to handling staplers in gastric cancer surgery. The limitations of this study were that it was conducted at a single institution and the sample size was 101 patients. Other researchers may need to perform the same study with a larger sample size to derive more accurate results. Accordingly, a long-term double-blind trial needs to be performed using well-matched groups of patients to confirm our findings.

## Data Availability

No datasets were generated or analysed during the current study.
